# Corrosion Mechanism and Electrochemical Reactions on Alloy 690 in Simulated Primary Coolant of Water–Water Energy Reactors

**DOI:** 10.3390/ma17081846

**Published:** 2024-04-17

**Authors:** Martin Bojinov, Iva Betova, Vasil Karastoyanov

**Affiliations:** 1 Department of Physical Chemistry, University of Chemical Technology and Metallurgy, 1756 Sofia, Bulgaria; vasko_kar@uctm.edu; 2 Institute of Electrochemistry and Energy Systems, Bulgarian Academy of Sciences, 1113 Sofia, Bulgaria; i.betova@iees.bas.bg

**Keywords:** alloy 690, primary water chemistry, electrochemical impedance spectroscopy, oxide growth, corrosion release, mixed-conduction model

## Abstract

During the power operation of the primary loop of a water cooled–water moderated energy reactor (WWER), the water chemistry evolves from a high-boron high-potassium composition to significantly lower concentrations of both constituents at the end of a campaign, and the Li concentration reaches ca. 0.7–0.9 ppm. In the present paper, the effect of primary water chemistry evolution during operation on the corrosion rate and conduction mechanism of oxides on Alloy 690 is studied by in situ impedance spectroscopy at 300 °C/9 MPa during 1-week exposures in an autoclave connected to a re-circulation loop. At the end of exposure, the samples were anodically polarized at potentials −0.8 to −0.1 V vs. SHE to evaluate the stability of the passive oxide. Simultaneously exposed samples of Alloy 690 were subsequently analyzed by XPS to estimate the thickness and in-depth composition of oxides. Impedance data were quantitatively interpreted using the mixed-conduction model (MCM) for oxide films. The effect of water chemistry evolution on the corrosion rate and conduction mechanism in the oxide on Alloy 690 in a primary coolant is discussed based on the obtained parameters.

## 1. Introduction

Most parts of pressurized water reactors (PWRs) use lithium hydroxide (LiOH) enriched in ^7^Li for primary side pH_T_ control (enriched LiOH). Naturally abundant lithium cannot be used, as it would generate an untenable increase in tritium production (a significant radioactive waste concern). Recently there have been interruptions in the enriched LiOH supply, resulting in significant concerns regarding the continued reliance on enriched LiOH. These concerns prompted industry evaluations of alternative production sources of enriched LiOH, recovery of ^7^Li from operating plants and an alternative chemical to enriched LiOH for pH_T_ control. Potassium hydroxide (KOH), which has been successfully used for pH_T_ control in WWERs for decades, is one potential alternative to enriched LiOH. Preliminary work was completed by the Electric Power Research Institute (EPRI) ca. 20 years ago, performing an initial assessment of potassium hydroxide as an alternative to lithium hydroxide for PWRs [1,2]. Although this work was initially focused on fuel-related concerns (namely, axial offset anomaly, AOA, now referred to as crud induced power shift, CIPS), it did identify several non-fuel-related issues that would need to be addressed and concluded that western PWRs could adopt KOH chemistry pending further investigation of the unknown effects. More recently, comprehensive research programs have been launched both by EPRI [3,4,5,6,7,8,9,10] and Framatome [11,12].

The prior literature of the effect of K on reactor internals, such as stainless steels, was reviewed in ref. [6] and it was concluded that the use of KOH is not expected to have a significant impact on the structural materials of the reactor. Based on a review of the literature, it appears the duplex oxide layers formed on stainless steels by LiOH in PWRs and KOH in WWERs are similar. In both types of systems, a chromium-depleted outer layer characterized by non-stoichiometric spinels forms over a chromium-rich inner layer. Test samples developed oxides between 0.28 and 0.66 μm thick after 5–6 months in the WWER primary coolant [6]. However, it is also noted that there is lack of specific data, particularly with respect to Ni-base alloys, and to the local chemistry that may develop in heated crevices, to occluded oxygenated environments.

The corrosion behavior of the main contemporary steam generator tube material for PWRs, namely, Alloy 690 (UNS N06690), has been extensively studied in the last decade in a simulated primary coolant [13,14,15,16,17,18,19,20,21,22,23,24,25,26,27,28,29,30]. The oxide films formed in nominal PWR conditions (~1000 ppm B, ~2 ppm Li, ~20–30 cm^3^ kg^−1^ dissolved H_2_) are usually described as a duplex oxide layer with a compact inner layer enriched in chromium and a discontinuous outer layer that contains mainly, according to the test facilities, nickel ferrite, nickel chromite and nickel hydroxide [14,16,26,29]. For passive films formed at the same temperature but at different anodic polarization potentials, the passive current density is independent of the formation potential, which is consistent with the n-type character of the barrier layer of the passive film, as evidenced by the modeling of impedance data using the point defect model (PDM) [21]. However, to the best of the authors’ knowledge, no experimental data on the oxide growth and corrosion release of this alloy in WWER primary chemistry have been reported, and also very limited data exist for B–Li ratios different to the nominal PWR primary coolant [30].

In this respect, the aim of the present paper is to quantify oxide formation and corrosion rates of Alloy 690 in a simulated WWER primary coolant at different stages of plant operation, and to compare the results to those obtained in nominal PWR primary chemistry. For this purpose, in situ chrono-potentiometric (corrosion potential vs. time) and current vs. potential curves were plotted, and electrochemical impedance spectroscopic (EIS) measurements were performed. The in-depth chemical composition of oxides was characterized by XPS. Kinetic and transport parameters of oxide growth and metal release were estimated by quantitative comparisons of the EIS data to the equations of the mixed-conduction model (MCM) for oxide films. Based on these, conclusions on the effect of water chemistry on oxide growth and corrosion release are drawn.

## 2. Materials and Methods

Samples were cut from Alloy 690 tubes (Special Metals) that were cold drawn, pickled and annealed at 1040 °C. The chemical compositions (both nominal and analyzed at a depth of 50 µm) are given in Table 1.

The working electrode pretreatment consisted of degreasing, electropolishing in 70% H_3_PO_4_-15%H_2_SO_4_-15%CH_3_OH at 9.0 V and 40 °C for 5 min and washing with de-ionized water. Experiments were performed at 300 ± 1 °C/9.0 ± 0.1 MPa in a stainless-steel autoclave (Parr, Moline, IL, USA) connected to a laboratory made re-circulation loop that allowed control of the electrolyte conductivity, pH and oxygen content with the appropriate sensors at room temperature. In particular, oxygen content was kept below 10 µg kg^−1^ throughout the experiments by continuous bubbling with N_2_ (99.999%). Electrochemical measurements were carried out in a three-electrode configuration featuring a Pt sheet (99.9%) counter electrode, and Pd (99.9%) sheet, cathodically polarized with a current of 10–30 µA vs. an additional Pt, as a reversible hydrogen reference electrode (RHE). The electrodes were mounted in a ceramic holder to ensure insulation and close proximity between the working and reference electrodes. All the potentials in the paper are re-calculated to the standard hydrogen electrode (SHE) scale. The electrolytes represented WWER primary water chemistries at three stages of operation—beginning-of-cycle (BOC), mid-cycle (MOC) and end-of-cycle (EOC), with the compositions being taken from actual in-line measurements during a specific campaign of a WWER-1000 reactor (Table 2). Nominal PWR water chemistry was also used for comparison. No NH_3_ was added to minimize the effect of hydrogen reactions on impedance response. One-week exposures to the respective coolants were followed by anodic polarization from −0.8 to −0.1 V vs. SHE to investigate the stability of the passive oxide. E_corr_ vs. time, current vs. potential and EIS measurements were implemented using an 10030 potentiostat (Ivium, Eindhoven, The Netherlands) in floating mode, frequency range of 11 kHz–0.1 mHz and AC amplitude of 50 mV. The linearity of the impedance spectra was verified by measuring with amplitudes from 20 to 60 mV, whereas causality was checked via compatibility with Kramers–Kronig transforms using the so-called measurement model and associated software [31]. All the experiments were repeated at least three times, and the reproducibility was better than ±1% per impedance magnitude and ±3° by phase angle.

XPS analyses of separate samples that were exposed in similar conditions were performed with an AXIS Supra apparatus (Kratos Analytical Ltd., Manchester, UK) using Al-Kα radiation (1486.6 eV). High resolution spectra were obtained with a pass energy of 20 eV, with the analyzed area being 1 mm^2^. The XPSPeak 4.1 software was employed for fitting the spectra using Gaussian–Lorentzian peaks (typical Lorentzian–Gaussian percentage of 60 ± 2%) after Shirley background subtraction. The relationship between sputtering time and depth was established based on the sputtering time of a Ta_2_O_5_ standard with known thickness (100 nm).

## 3. Results

### 3.1. Corrosion Potential vs. Time and Voltammetric Measurements

**Figure 1:**
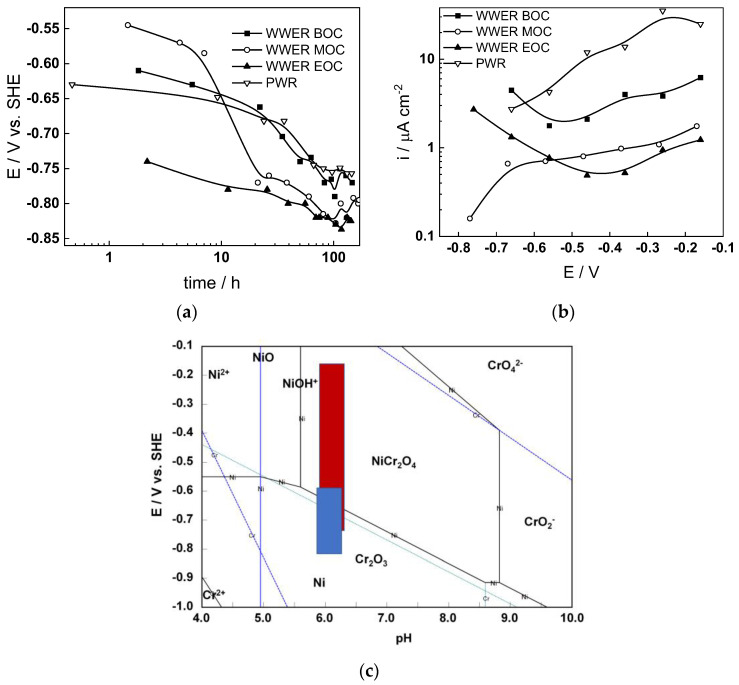
Corrosion potential vs. time (**a**) and current vs. potential (**b**) curves and the span of corrosion potential values (blue) and anodic polarization potentials (red) shown in an E–pH diagram of the Ni-Cr-H_2_O system at 300 °C/9 MPa (**c**).

The corrosion potential (E_corr_) vs. time curves of Alloy 690 in the four studied environments are summarized in Figure 1a. The E_corr_ decreases logarithmically with time, indicating the transformation of the electropolished surface into a corrosion film, and reaches constant values after 40–50 h. The rate of decrease seems to be the fastest in WWER MOC chemistry, and the least expressed in WWER EOC chemistry. The interval of E_corr_ values shown with a blue rectangle in the E–pH diagram of the Ni-Cr-H_2_O system (Figure 1c) is located in the Cr_2_O_3_ + Ni(0) stability region, i.e., most probably chromium oxide with some incorporated nickel is formed. Current vs. potential curves during the anodic polarization of samples after a week of exposure to the respective water chemistry are collected in Figure 1b. A current increase is observed in the middle of the studied range of potentials, probably indicating a gradual transformation of (Cr, Ni)_2_O_3_ oxide to NiCr_2_O_4_ as corroborated by the E–pH diagram—intersection of the stability line of Cr_2_O_3_ + Ni and NiCr_2_O_4_ with the superimposed potential range of anodic polarization (red rectangle) coincides with the current increase. It is worth mentioning that the current densities are the largest in the nominal PWR, followed by the WWER BOC (i.e., the two water chemistries in which boric acid concentration is the highest). On the other hand, current densities in the WWER MOC and EOC chemistries are significantly lower. Thus, it can be stated that the stability of the oxide during anodic polarization is related to boric acid concentration in the electrolyte.

### 3.2. Electrochemical Impedance Spectroscopy

**Figure 2:**
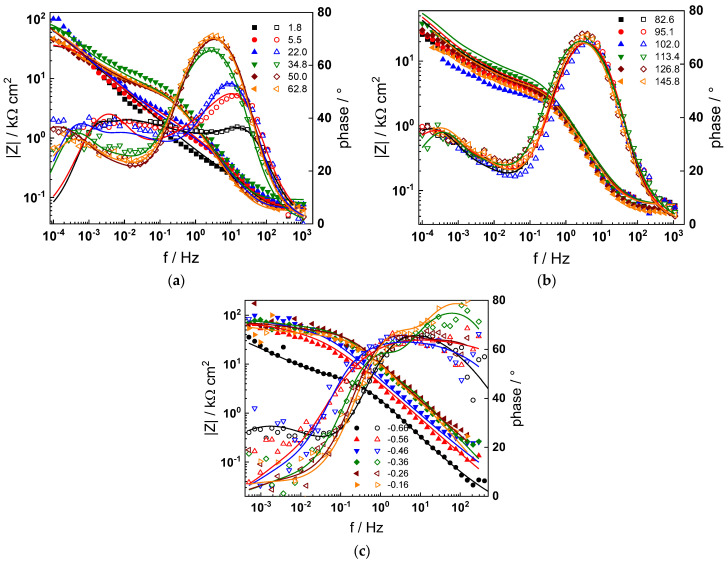
Impedance spectra at the corrosion potential (**a**) at 1.8–63 h, (**b**) at 82–146 h and (**c**) under anodic polarization in WWER BOC chemistry. Closed symbols—impedance magnitude vs. frequency, open symbols—phase shift vs. frequency. Points—experimental values, solid lines—best-fit calculation according to the proposed model.

**Figure 3:**
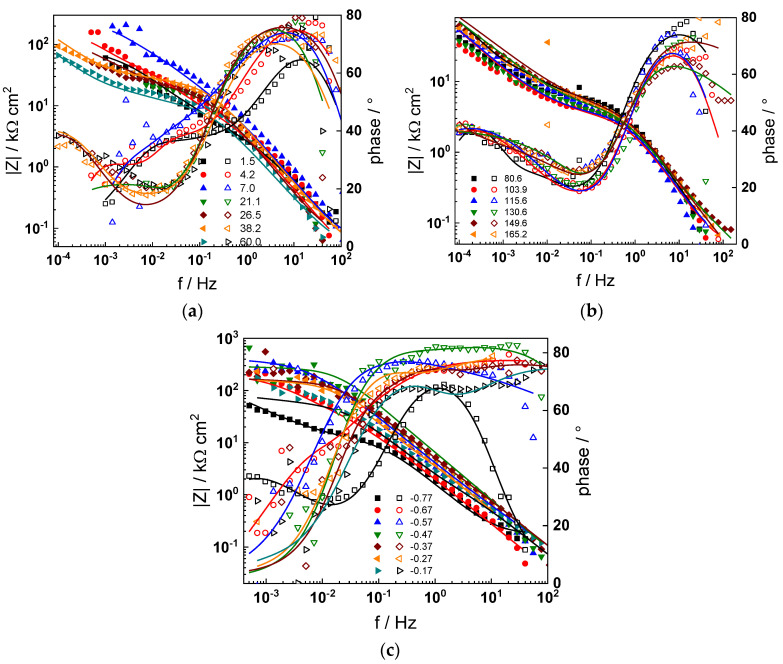
Impedance spectra at the corrosion potential (**a**) at 1.5–60 h, (**b**) at 81–165 h, and (**c**) under anodic polarization in WWER MOC chemistry. Closed symbols—impedance magnitude vs. frequency, open symbols—phase shift vs. frequency. Points—experimental values, solid lines—best-fit calculation according to the proposed model.

**Figure 4:**
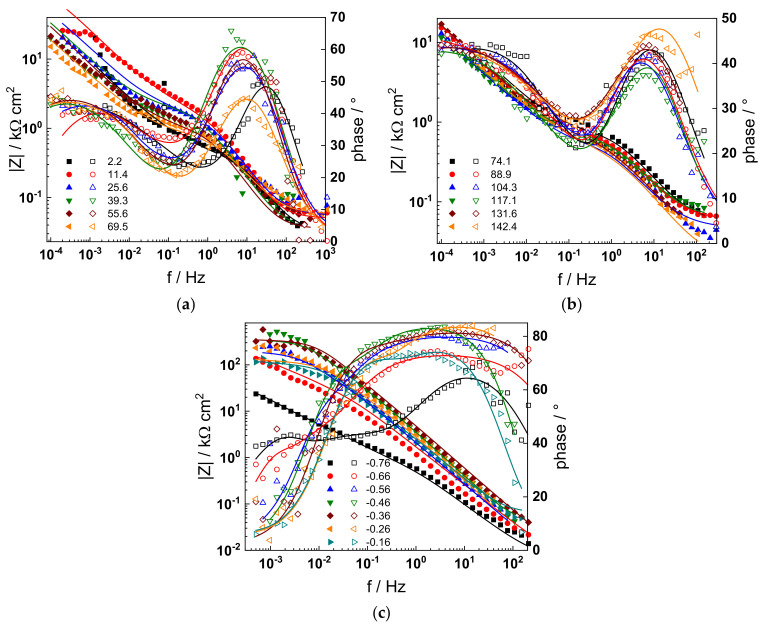
Impedance spectra at the corrosion potential (**a**) at 1.5–70 h, (**b**) at 74–142 h, and (**c**) under anodic polarization in WWER EOC chemistry. Closed symbols—impedance magnitude vs. frequency, open symbols—phase shift vs. frequency. Points—experimental values, solid lines—best-fit calculation according to the proposed model.

**Figure 5:**
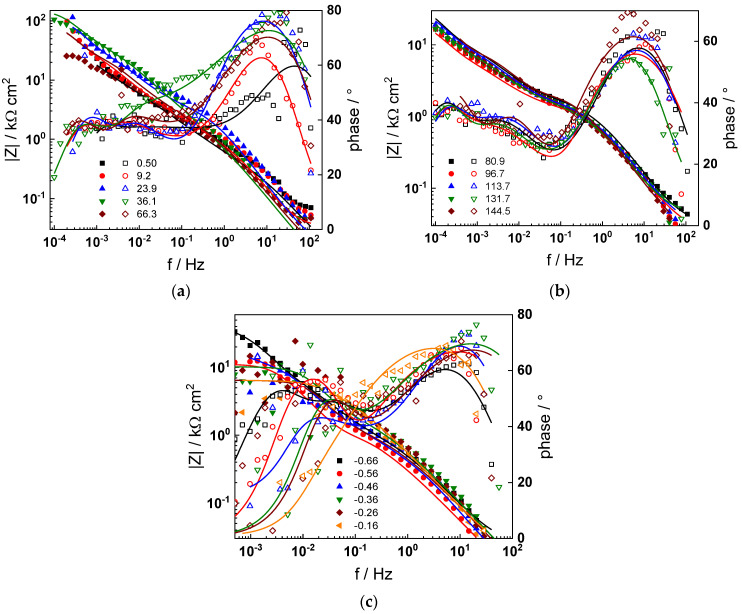
Impedance spectra at the corrosion potential (**a**) at 1.5–66 h, (**b**) at 80–144 h and (**c**) under anodic polarization in PWR chemistry. Closed symbols—impedance magnitude vs. frequency, open symbols—phase shift vs. frequency. Points—experimental values, solid lines—best-fit calculation according to the proposed model.

The electrochemical impedance spectra of Alloy 690 at the corrosion potential in the four studied primary coolant chemistries are collected in Figure 2a,b, Figure 3a,b, Figure 4a,b and Figure 5a,b. The respective spectra measured during anodic polarization after a week of exposure are shown in Figure 2c, Figure 3c, Figure 4c and Figure 5c. The impedance magnitude at f→0, that can be approximated to the polarization resistance, first decreases, then increases slowly with time, indicating the transformation of the pre-treatment layer into a passive oxide followed by further growth of the latter. The evolution of impedance in the WWER EOC is slower in comparison to the WWER BOC—the steady-state |Z|_f→0_ values are reached after ca. 80 h in EOC, whereas in BOC, this takes ca. 60 h. The evolution of impedance in PWR chemistry is also slower than in the WWER BOC and |Z|_f→0_ values are somewhat smaller than those in WWER chemistry, indicating higher oxidation and corrosion release rates. Concerning the evolution of impedance during anodic polarization, the impedance magnitude at low frequencies increases significantly for potentials above the Ni(0)→Ni(II) transition, i.e., it produces a layer that appears more corrosion resistant. Once again, in agreement with the current vs. potential curves, the |Z|_f→0_ values measured in the PWR are considerably lower, indicating lower stability of the oxide in this medium when compared to the WWER coolant chemistries.

Using the distribution of relaxation times method [32], five time constants were detected in the spectra. They are hypothesized to reflect the electronic properties of the oxide, a two-step charge transfer at the oxide/coolant interface and diffusion-migration of two types of defects through the oxide. The differences between the frequency ranges of the respective time constants in different coolant chemistries are comparatively small at the open-circuit potential. Deconvolution of spectra measured under anodic polarization did not reveal any extra time constants; the only notable difference observed above the Ni(0)–Ni(II) transition being the shift of all time constants towards higher frequencies. This feature can in principle be explained by a somewhat faster ionic transport in the oxide formed at higher potentials, which seems to be, however, more than compensated by the decrease in the rate of interfacial reactions leading to an overall significant increase of the polarization resistance.

### 3.3. Chemical Analysis of Oxides

**Figure 6:**
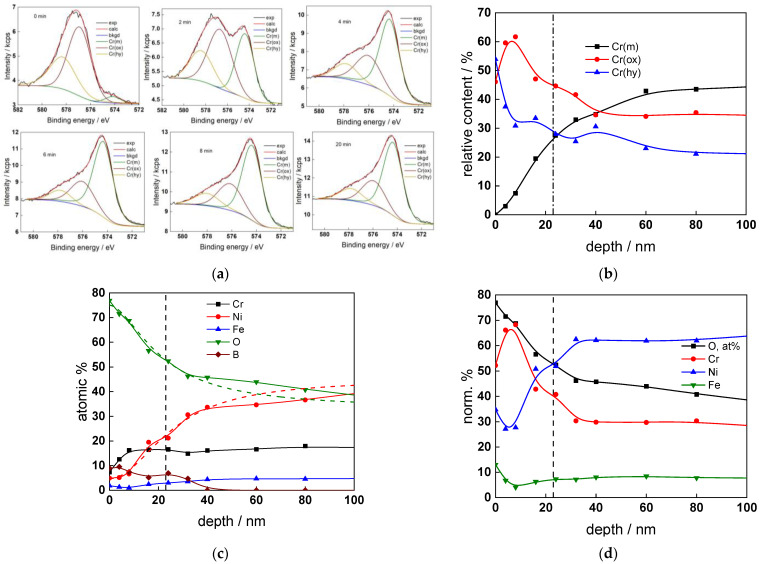
XPS analysis of the oxide formed in the WWER BOC: (**a**) Cr2p spectra as a function of etching time, (**b**) distribution of oxidation states of Cr vs. depth, (**c**) atomic concentrations of oxide components vs. depth, (**d**) normalized cation content of oxide vs. depth. Sigmoidal fits to oxygen and nickel profiles shown with dashed lines.

**Figure 7:**
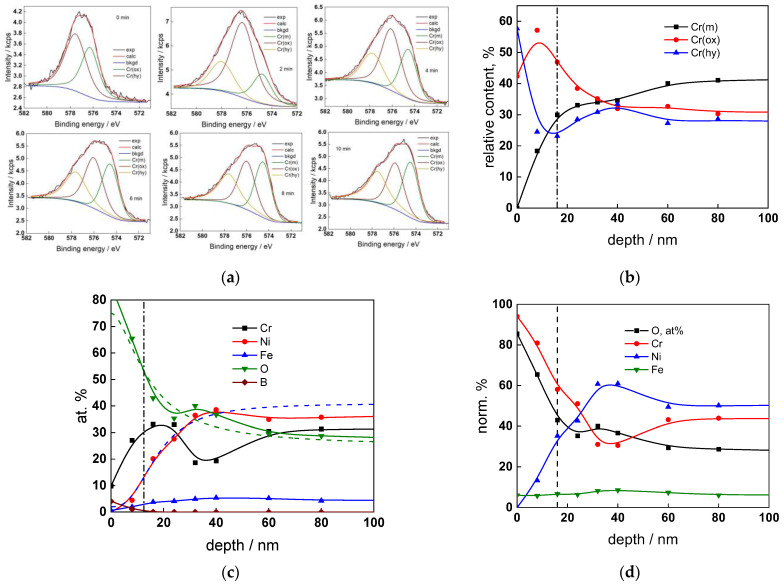
XPS analysis of the oxide formed in the WWER MOC: (**a**) Cr2p spectra as a function of etching time, (**b**) distribution of oxidation states of Cr vs. depth, (**c**) atomic concentrations of oxide components vs. depth, (**d**) normalized cation content of oxide vs. depth. Sigmoidal fits to oxygen and nickel profiles shown with dashed lines.

**Figure 8:**
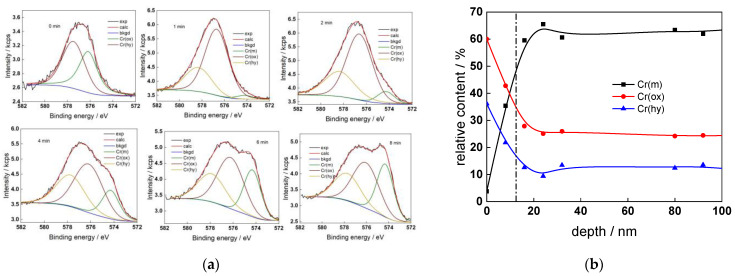

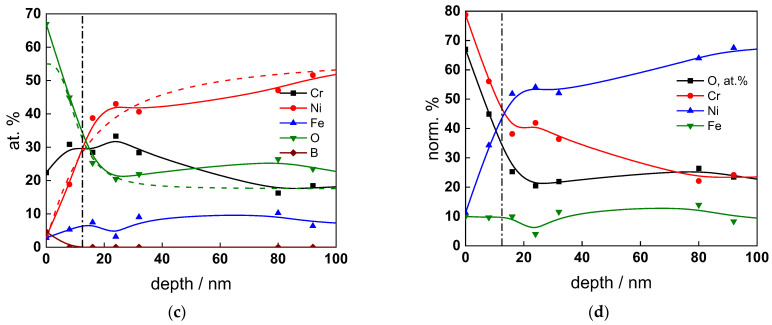
XPS analysis of the oxide formed in the WWER EOC: (**a**) Cr2p spectra as a function of etching time, (**b**) distribution of oxidation states of Cr vs. depth, (**c**) atomic concentrations of oxide components vs. depth, (**d**) normalized cation content of oxide vs. depth. Sigmoidal fits to oxygen and nickel profiles shown with dashed lines.

**Figure 9:**
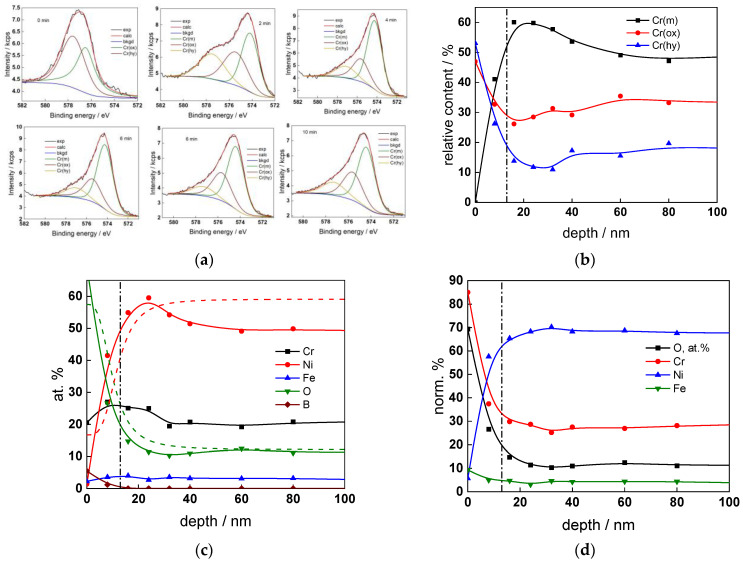
XPS analysis of the oxide formed in the PWR: (**a**) Cr2p spectra as a function of etching time, (**b**) distribution of oxidation states of Cr vs. depth, (**c**) atomic concentrations of oxide components vs. depth, (**d**) normalized cation content of oxide vs. depth. Sigmoidal fits to oxygen and nickel profiles shown with dashed lines.

The chemical composition of the oxides was assessed by XPS depth profiling. Detailed Cr2p spectra, as well as distributions of Cr oxidation states with depth, atomic concentrations of main constituents and normalized cation content with depth are collected in Figure 6, Figure 7, Figure 8 and Figure 9, depending on the coolant chemistry. The position of the oxide/alloy interface has been averaged from sigmoidal fits to the oxygen and nickel profiles and the depth at which the relative content of metallic Cr becomes higher than the sum of the two oxidized states—Cr(III) oxide and hydroxide, respectively, as in our previous work [30]. The oxide layers formed are rather thin (12–23 nm), the thickest layer being formed in the WWER EOC water chemistry, and no evidence of a bilayer structure was found, contrary to long-term exposure results discussed in the literature [14,18,26,29]. Cr is the main component of the oxides, accounting for more than 60% of the normalized cation content. A Cr depletion zone is observed below the oxides. Boron is found at the coolant/oxide interface in all cases, whereas Li was difficult to quantify due to the overlap of the Li1s region with the Fe3p and Ni3p regions in the XP spectra. No potassium was found either in the oxide or at the interface. Summarizing, very thin passive layers are formed on Alloy 690, which most probably explains the well-detected interfacial reactions and their important role in the corrosion mechanism. A rationalization of this mechanism is attempted in the next section. 

## 4. Discussion

### Kinetic Model

To quantitatively interpret the obtained experimental data, a kinetic model employed previously for the corrosion mechanism of Alloy 690 in secondary side water chemistry is employed [33]. Briefly, the process of chromium oxide layer growth on Alloy 690 via Cr oxidation at the metal/film interface and oxygen ingress by a vacancy mechanism is represented by the reaction sequence:

$$\begin{array}{l} {\mathrm{Cr}}_{m}\overset{k_{O}}{\to }{\mathrm{Cr}}_{\mathrm{Cr}}+1.5V_{O\left(M/F\right)}^{••}+3e_{m}^{-}\\ 1.5V_{O\left(M/F\right)}^{••}\overset{D_{O},\vec{E}}{\to }1.5V_{O\left(F/S\right)}^{••}\\ 1.5V_{O\left(F/S\right)}^{••}+1.5H_{2}O\overset{k_{2O}}{\to }1.5O_{O}+3H^{+} \end{array}$$

Oxide growth is balanced by its chemical dissolution to achieve steady state thickness:

$${{(\mathrm{Ni}}_{x}{\mathrm{Cr}}_{1-x})}_{2}O_{3}+6H^{+}\overset{k_{d}}{\to }2x{\mathrm{Ni}}^{2+}+2(1-x){\mathrm{Cr}}^{3+}+3H_{2}O$$

Dissolution of cations such as Ni or Fe through chromium oxide, i.e., corrosion release, is governed by the oxidation of the metal, transport of its ions by indirect interstitial mechanisms and ejection of a cation in the coolant:

$$\begin{array}{l} {\mathrm{Ni}}_{m}\overset{k_{M}}{\to }{\mathrm{Ni}}_{i\left(M/F\right)}^{••}{+2e}_{m}^{-}\\ {\mathrm{Ni}}_{i\left(M/F\right)}^{••}\overset{D_{M},\vec{E}}{\to }{\mathrm{Ni}}_{i\left(F/S\right)}^{••}\\ {\mathrm{Ni}}_{i\left(F/S\right)}^{••}\overset{k_{2M}}{\to }{\mathrm{Ni}}_{\mathrm{aq}}^{y+}+(y-2)e_{m}^{-} \end{array}$$

The rate constants at the respective interfaces *k*_1*O*_, *k*_1*M*_, *k*_2*O*_ and *k*_2*M*_, are assumed to obey an exponential dependence on the applied potential:

$$k_{1M}={k_{1M}}^{0}e^{\frac{3\alpha_{1M}\left(1-\alpha \right)F}{RT}E},\;k_{1O}={k_{1O}}^{0}e^{\frac{3\alpha_{1O}\left(1-\alpha \right)F}{RT}E},\;k_{2M}={k_{2M}}^{0}e^{\frac{\alpha_{2M}\alpha F}{RT}E},\;k_{2O}={k_{2O}}^{0}e^{\frac{\alpha_{2M}\alpha F}{RT}E}$$

At open-circuit potential, corrosion proceeds due to the consumption of electrons produced by the oxidation reactions by the reduction of water. The process at the film/solution interface is most probably dominated by the water reduction-hydrogen oxidation reaction that is assumed to obey a Volmer–Heyrovsky mechanism:

$$\begin{array}{l} H_{2}{O+e}^{-}\overset{k_{1H}}{\underset{k_{-1H}}{⇄}}H_{\mathrm{ad}}{(\theta )+\mathrm{OH}}^{-}\\ H_{\mathrm{ad}}{(\theta )+H}_{2}{O+e}^{-}\overset{k_{2H}}{\underset{k_{-2H}}{⇄}}H_{2}{+\mathrm{OH}}^{-} \end{array}$$

with the respective rate constants depending exponentially on the potential

$$k_{\pm iH}={k_{\pm iH}}^{0}e^{\pm b_{\pm iH}E},\;b_{\pm iH}=\frac{\alpha_{\pm iH}\alpha F}{RT}$$

The impedance of the *F/S* interface is derived in Ref. [33]

$$Z_{F/S}=\frac{1}{Z_{f,F/S}^{-1}+j\omega C_{F/S}}$$

The capacitance *C_F/S_* is generalized as a constant phase element expressing geometrical and/or energetical heterogeneity of the interface. The faradaic impedance *Z_f, F/S_* is given by:

$$Z_{f,F/S}^{-1}=R_{F/S}^{-1}+\frac{AX}{Z_{H}+j\omega \beta }$$

where

$$\begin{array}{l} R_{F/S}^{-1}=F\left(k_{-1H}b_{-1H}+k_{2H}b_{2H}\right)\bar{\theta }+F\left(k_{2H}b_{2H}+k_{1H}b_{1H}+(y-2)k_{2M}b_{2M}\right)\left(1-\bar{\theta }\right)\\ A=F\left(k_{1H}+k_{-1H}-k_{-2H}-k_{2M}-k_{2}\right)\\ X=k_{-2H}b_{-2H}-\left(k_{-2H}b_{-2H}-k_{1H}b_{1H}+k_{-1H}b_{-1H}-k_{2H}b_{2H}\right)\bar{\theta }-k_{1H}b_{1H}\\ \bar{\theta }=\frac{k_{2H}+k_{1H}}{Z_{H}\;},\;Z_{H}=k_{-2H}+k_{1H}+k_{-1H}+k_{2H} \end{array}$$

Adopting the transport equations of point defects of the MCM in the low field limit that predominates in oxides formed in high-temperature water [30], and with some assumptions to simplify the mathematical expressions, the impedance of transport of oxygen vacancies is of the form

$$Z_{ion,O}\approx \frac{RT}{4F^{2}k_{O}(1-\alpha )\left(1+\sqrt{1+\frac{4j\omega }{D_{O}K^{2}}}\right)},K=\frac{F}{RT}\vec{E}$$

The impedance of transport of interstitial cations is derived analogously as

$$Z_{ion,M}=\frac{RT}{4F^{2}k_{M}(1-\alpha )\left[1+\sqrt{1+\frac{j\omega }{D_{M}K^{2}}}\right]}$$

Here, *α* is the part of the potential consumed as a drop at the film/solution interface (assumed to be equal to 0.85 [30]), $\vec{E}$ is the field strength in the chromium oxide, and *D_O_* and *D_M_* are diffusivities of oxygen and metal cations. Since ionic point defects play the role of electron donors and their concentration depends both on potential and the distance within the oxide, the impedance of the electronic properties of the oxide reads as:

$$Z_{e}\approx \frac{RT}{2j\omega F\vec{E}L_{O}C_{sc}}\ln\frac{\left[1+j\omega \frac{RT}{F^{2}D_{e}}\left(\frac{k_{2O}}{k_{O}}+\frac{k_{2M}}{k_{M}}\right)\varepsilon \varepsilon_{0}\exp\left(2\frac{F\vec{E}}{RT}L_{O}\right)\right]}{1+j\omega \frac{RT}{F^{2}D_{e}}\left(\frac{k_{2O}}{k_{O}}+\frac{k_{2M}}{k_{M}}\right)\varepsilon \varepsilon_{0}}$$

where *L_O_* is the oxide film thickness

$$L_{O}=L_{t=0}+\frac{1}{b}\ln\left[1+V_{m,\mathrm{MO}}k_{1O}be^{-bL_{t=0}}t\right],\;b=\frac{3\alpha_{1O}F\vec{E}}{RT}$$

As usual, ε is the dielectric constant of the chromium oxide film (a value of 25 was adopted in analogy to previous work [30]), *D_e_* the diffusivity of electrons and *C_sc_* the depletion layer capacitance. Finally, the total impedance is written as

$$Z=R_{ohm}+Z_{f}+Z_{F/S}=R_{ohm}+{\left(Z_{e}^{-1}+Z_{ion,O}^{-1}+Z_{ion,M}^{-1}\right)}^{-1}+Z_{F/S}$$

**Figure 10:**
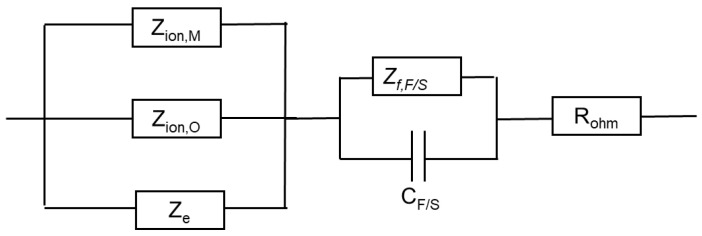
Equivalent electrical circuit illustrating the connections between impedance components.

An equivalent circuit that illustrates the connections between different impedances is shown in Figure 10.

**Figure 11:**
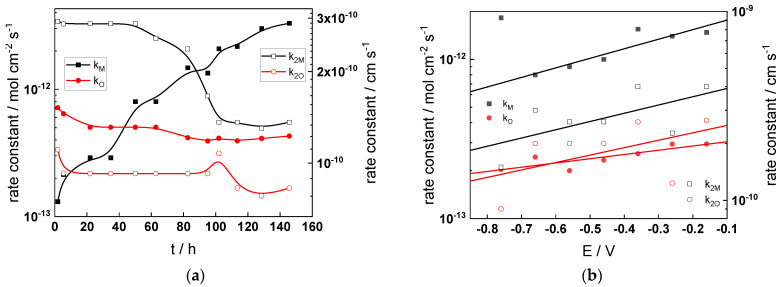

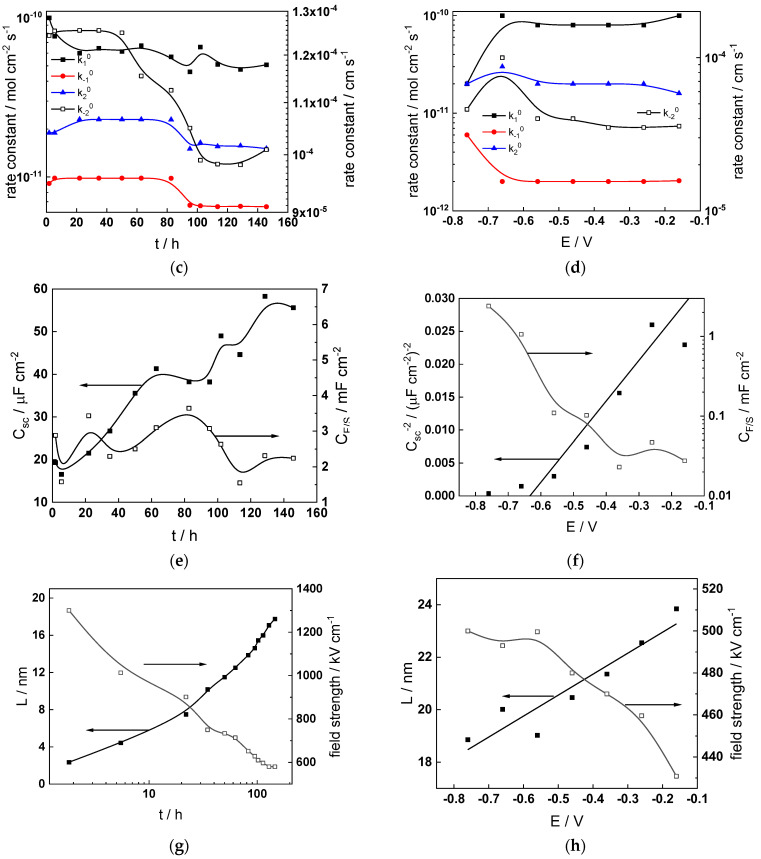
Model parameters as a function of time (**a**,**c**,**e**,**g**) and potential (**b**,**d**,**f**,**h**) for Alloy 690 in the WWER BOC: (**a**,**b**) rate constants of oxide formation and metal dissolution, (**c**,**d**) rate constants of the hydrogen reactions, (**e**,**f**) capacitances of the space charge layer and the film/solution interface and (**g**,**h**) film thickness and field strength in the oxide.

**Figure 12:**
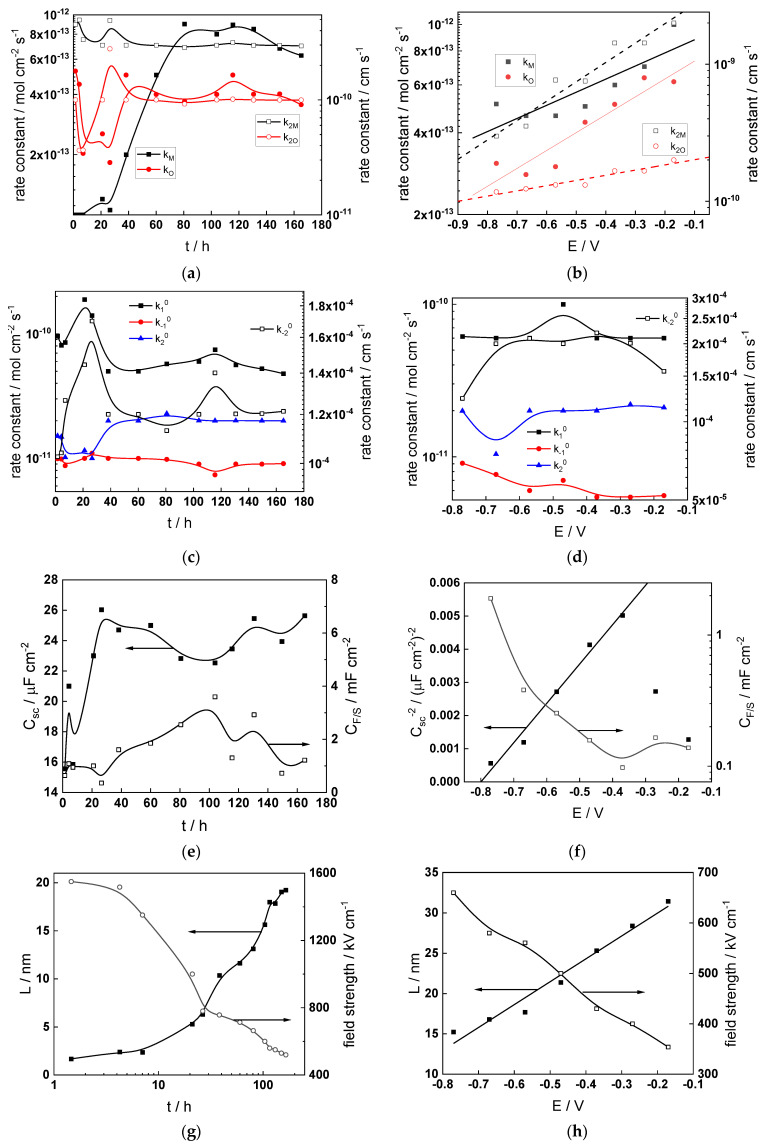
Model parameters as a function of time (**a**,**c**,**e**,**g**) and potential (**b**,**d**,**f**,**h**) for Alloy 690 in the WWER MOC: (**a**,**b**) rate constants of oxide formation and metal dissolution, (**c**,**d**) rate constants of the hydrogen reactions, (**e**,**f**) capacitances of the space charge layer and the film/solution interface and (**g**,**h**) film thickness and field strength in the oxide.

**Figure 13:**
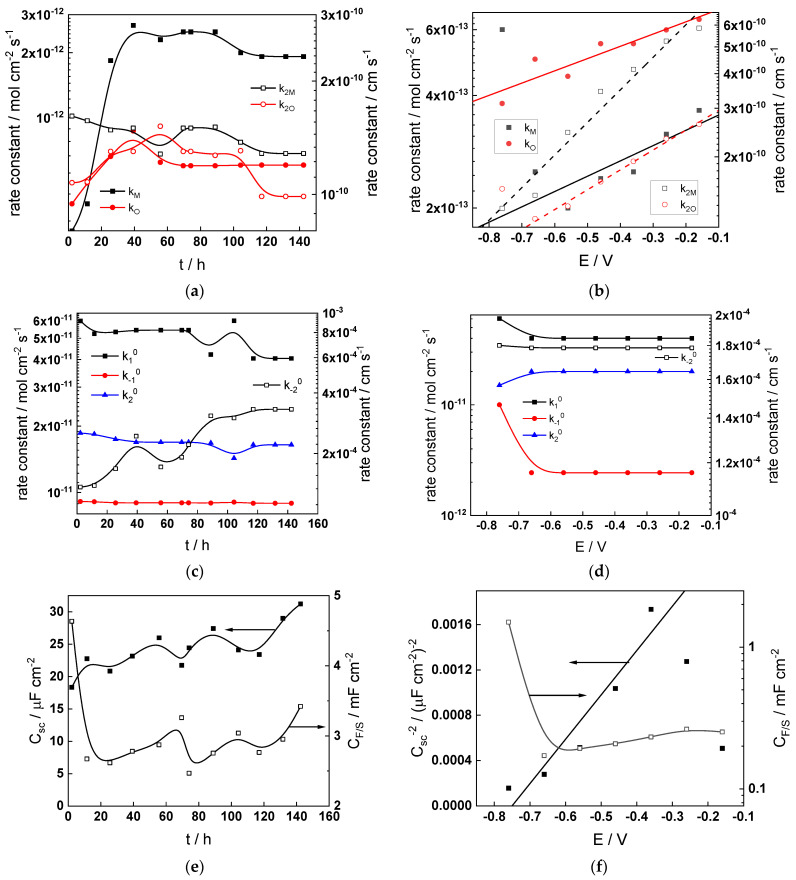

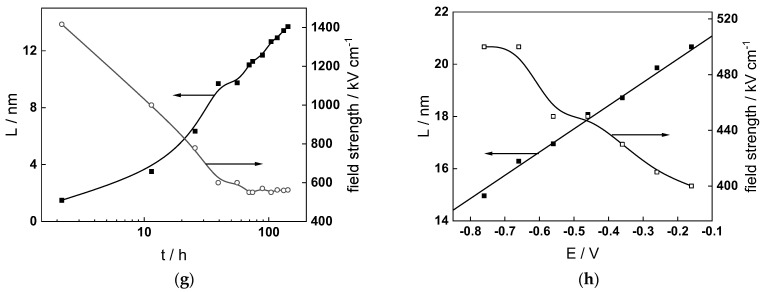
Model parameters as a function of time (**a**,**c**,**e**,**g**) and potential (**b**,**d**,**f**,**h**) for Alloy 690 in the WWER EOC: (**a**,**b**) rate constants of oxide formation and metal dissolution, (**c**,**d**) rate constants of the hydrogen reactions, (**e**,**f**) capacitances of the space charge layer and the film/solution interface and (**g**,**h**) film thickness and field strength in the oxide.

**Figure 14:**
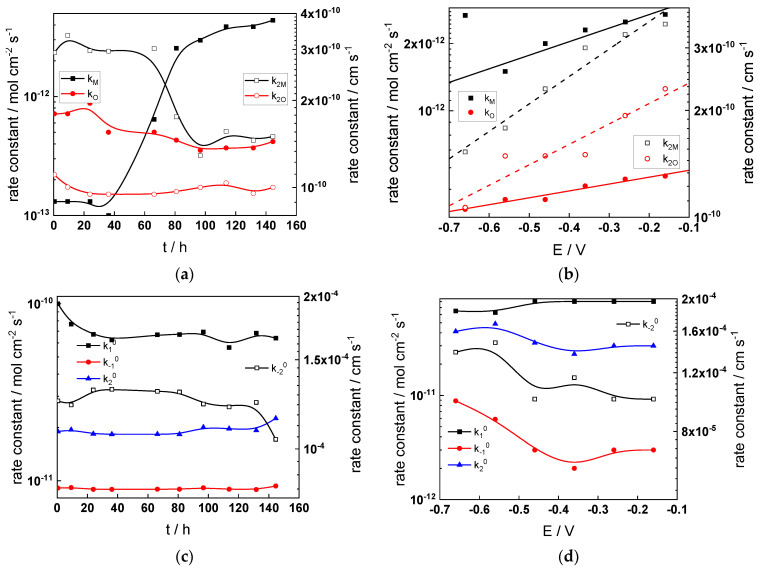

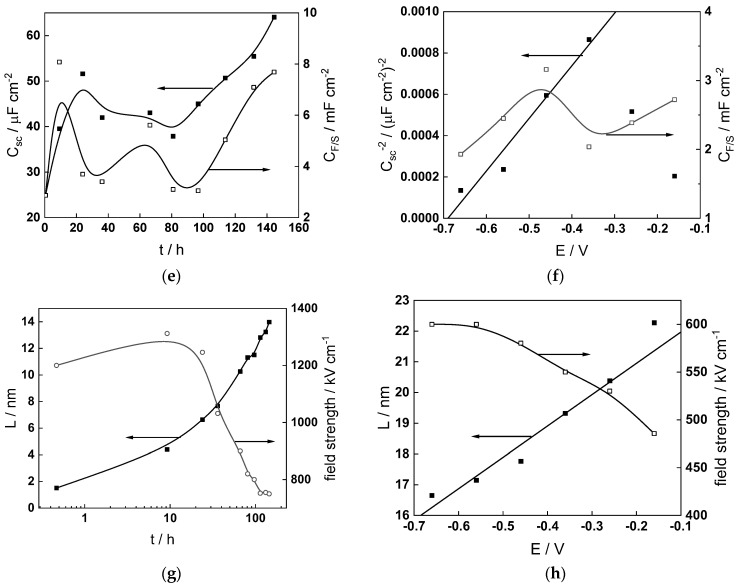
Model parameters as a function of time (**a**,**c**,**e**,**g**) and potential (**b**,**d**,**f**,**h**) for Alloy 690 in the PWR: (**a**,**b**) rate constants of oxide formation and metal dissolution, (**c**,**d**) rate constants of the hydrogen reactions, (**e**,**f**) capacitances of the space charge layer and the film/solution interface and (**g**,**h**) film thickness and field strength in the oxide.

Parameterization of the model was achieved via a complex fitting of the impedance data to the transfer function using the Levenberg–Marquardt algorithm. To decrease the number of fitting parameters, all the transfer coefficients of steps involved in the hydrogen reaction were assumed to be equal to 0.5. Solid lines in Figure 2, Figure 3, Figure 4 and Figure 5 illustrate the ability of the model to reproduce the impedance response for all the studied conditions. The dependences of model parameters on time and applied potential are collected in Figure 11, Figure 12, Figure 13 and Figure 14 as a function of coolant chemistry, whereas the values of parameters that are independent of time and/or potential are summarized in Table 3. 

The following conclusions can be drawn from the parameter values:

- The rate constants at the alloy/oxide interface evolve with time in opposite directions—the rate constant of oxide formation, k_O_, decreases with time, whereas that of nickel oxidation to produce interstitial cations, k_M_, increases. This is most probably due to the process of transformation of the pre-treatment layer to a passive film and indicates that the chromium oxide formation reaction is faster at the alloy/pre-treatment layer interface, with the opposite holding true for the nickel oxidation process. At any rate, both constants stabilize after 60–80 h indicating that the passive film reaches a quasi-steady state. Concerning the effect of water chemistry on interfacial rate constants, the largest values of k_M_ are observed in the PWR, whereas the smallest are in the WWER MOC.
- The dependences of the interfacial rate constants on potential derived from the measurements under anodic polarization are exponential but rather weak (transfer coefficients of 0.08–0.20), i.e., the kinetic barriers at both interfaces are rather asymmetric and the transition state is rather similar to the initial state, as already found before for iron and stainless steels [34,35].
- The rate constants of the hydrogen reactions at the film/solution interface also stabilize after 60–80 h of exposure, their extent of evolution with time being much smaller in comparison to the oxide formation and corrosion release processes. The differences in the rate constant values estimated from experiments in different water chemistries indicate that the effect of water chemistry is rather small, which is understandable given the comparatively small variations in oxide thickness and composition and the fact that pH of all solutions is almost constant (the rates of water reduction and hydrogen evolution are expected to depend mainly on pH). It is worth mentioning that the kinetic parameters of hydrogen reactions are somewhat different under anodic polarization, corroborating the hypothesis of a different nature of the oxide at open circuit and under polarization. However, further experiments at different pH values are needed to confirm or discard this hypothesis.

- It is evident that the diffusivities of electrons, oxygen and metal cations are hardly dependent on water chemistry, in agreement with the small variations in oxide film composition. It is noteworthy that the diffusion coefficients of ionic carriers in the film formed during anodic polarization are larger than those at open circuit, whereas the opposite is true for the diffusion coefficient of electrons. This once again points out the different nature of oxides formed at the corrosion potential and under anodic bias.
- The field strength in the oxide in general decreases with time both at open circuit and during anodic polarization. One way to interpret this dependence is to consider the formation of a space charge in the oxide due to the very large differences between the rates of ionic and electronic transport during its growth. Assuming that the total defect concentration to create the space charge is the vectorial sum of the concentrations of the two mobile carriers—oxygen vacancies and interstitial cations—and immobile charges due to the incorporation of nickel at an oxidation state lower than 3 in chromium oxide, the generalized expression for the field strength has the form [36]

$$\vec{E}(L)={\vec{E}}_{0}+\frac{e}{\varepsilon \varepsilon_{0}}\int_{0}^{L}\Delta c(x)dx,\;\Delta c(x)=\left[2c_{o}(x)-c_{e^{\prime }}(x)-z_{Ni}c_{Ni}(x)\right]$$

- Treating the defect concentration as homogeneous for simplicity, we arrive at

$$\vec{E}(L)={\vec{E}}_{0}+L\frac{F\Delta c(0)}{\varepsilon \varepsilon_{0}\left(1+\frac{L}{x_{0}}\right)},x_{0}=\frac{\varepsilon \varepsilon_{0}RT}{{\left(2F\right)}^{2}ac_{o}(0)}$$

**Figure 15:**
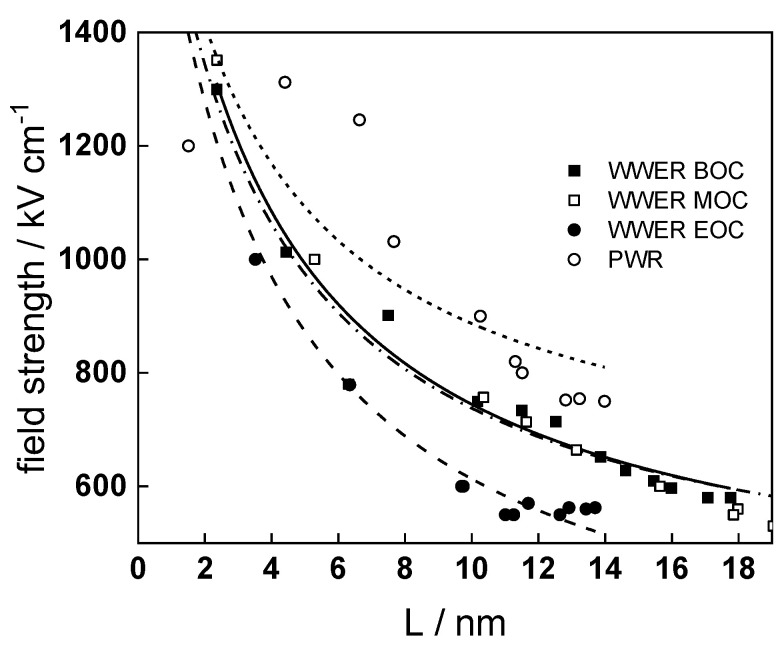
Field strength vs. oxide thickness dependences interpreted by the space charge approximation outlined in the text.

Fits of the field strength vs. oxide thickness dependences using this equation are illustrated in Figure 15 and demonstrate its ability to reproduce the experimental data. The estimated values of *Δc(0)* are of the order of 10^−4^ mol cm^−3^, whereas those of *x_0_* are ca. 3–4 nm, inherently reasonable for such thin and defective oxides. 

Summarizing, it can be stated that since the lowest corrosion and oxidation rates are observed in the WWER MOC, and the highest in the nominal PWR and WWER BOC, boric acid and to certain extent LiOH have a negative effect on these rates. This indicates that during a significant part of a WWER campaign, the corrosion and oxidation rates of Alloy 690 will be lower than those in a nominal PWR. Of course, this conclusion is preliminary and awaits more experiments in solutions with different pH and temperature to encompass all the relevant operating conditions. In addition, the hypothesis concerning the transformation of the barrier layer on the alloy upon anodic polarization needs further verification via surface analysis of oxides formed at different potentials. Such measurements are planned to be performed in the near future. 

## 5. Conclusions

We report a characterization of the corrosion mechanism of the main steam generator tube material of PWRs—Alloy 690—in WWER primary water chemistry by in situ impedance spectroscopy complemented with ex situ analysis of oxides with XPS. Measurements in nominal PWR chemistry are performed and analyzed for comparison. The mechanism is modeled using a new version of the mixed-conduction model featuring a two-step hydrogen reaction at the film/solution interface. The following conclusions can be drawn from the experimental data and calculation results:

- The effect of water chemistry on the conduction mechanism on the growing oxide, corrosion release and electrochemical reactions is in general small, indicating that no general corrosion issues are expected for Alloy 690 during an eventual transition from B-Li to B-K-Li primary water chemistry.
- It can be concluded that higher oxidation and corrosion release rates are observed in the WWER BOC and nominal PWR chemistries, i.e., compositions with the highest content of boric acid. Thus, it can be presumed that boric acid has a certain accelerating effect on corrosion. This is to a certain extent correlated with the adsorption or incorporation of B in the outermost layers of the oxide, even if the extent of incorporation does not markedly depend on boric acid concentration. Since the lower corrosion rate is observed in the WWER MOC, it can be also tentatively concluded that the Li concentration in the coolant has an adverse effect on corrosion.
- On the other hand, anodic polarization of oxides formed after a week of exposure leads to the transformation of the oxide, most probably from the (Cr, Ni)_2_O_3_ to the NiCr_2_O_4_ type. This leads to a decrease of the rate of electronic conduction and an increase of the ionic conduction rate through the film; the effect being the most pronounced for layers formed in nominal PWR chemistry. This result indicates that passivation of Alloy 690 in B-K-Li chemistry is more efficient and creates an oxide that is less susceptible to corrosion during an increase of the redox potential, e.g., during ingress of oxygen in the system. This finding has potential implications in relation to the development of localized corrosion modes and will be studied in more detail in future work.

## Figures and Tables

**Table 1:** Nominal and analyzed compositions of the sample material (Alloy 690).

Content, wt%	C	Fe	Cr	Cu	Mn	Ni	Al	S	Si	Mo
nominal	≤0.03	9.0–10.0	29.0–31.0	0.05	0.10	Bal.	≤0.50	≤10^−4^	0.10	0.15
analyzed	0.025	9.1	29.5	0.03	0.07	Bal.	0.17	n.d.	0.14	0.14

**Table 2:** Compositions of the primary coolant used in the investigation.

Coolant	H_3_BO_3_/g kg^−1^	KOH/mg kg^−1^	LiOH/mg kg^−1^	pH (25 °C)
WWER, beginning-of-cycle (BOC)	1.20	11.0	0.1	6.0
WWER, mid-cycle (MOC)	0.80	7.0	0.5	6.1
WWER, end-of-cycle (EOC)	0.40	3.5	0.9	6.2
PWR, nominal	1.20	0.0	1.0	5.9

**Table 3:** Parameters that did not depend on time or applied potential.

Water chemistry/ Parameter	10^8^ D_e_/ cm^2^ s^−1^	10^17^ D_M_/ cm^2^ s^−1^	10^17^ D_O_/ cm^2^ s^−1^	α*_M_*	α*_O_*	α_2*M*_	α_2*O*_
**WWER BOC** E_corr_	0.20	0.40	0.20	-	-	-	-
**WWER BOC** Anodic region	1.0	4.0	2.0	0.16	0.20	0.13	0.13
**WWER MOC** E_corr_	1.1	0.30	0.20	-	-	-	-
**WWER MOC** Anodic region	0.80	3.8	1.8	0.11	0.16	0.18	0.10
**WWER EOC** E_corr_	3.5	0.40	0.10	-	-	-	-
**WWER EOC** Anodic region	0.10	3.0	0.50	0.10	0.090	0.15	0.10
**PWR** E_corr_	5.0	0.8	0.2	-	-	-	-
**PWR** Anodic region	2.0	3.0	2.0	0.15	0.080	0.10	0.08

## Data Availability

Data are contained within the article.
